# Supplementary material for: The Effects of Crosslinking on the Rheology and Cellular Behavior of Polymer-Based 3D-Multilayered Scaffolds for Restoring Articular Cartilage
Source: Polymers (Basel). 2021 Mar 16;13(6):907. doi: 10.3390/polym13060907 (PMC7999668; doi:10.3390/polym13060907)
Supplement: Supplementary file 1 [file polymers-13-00907-s001.pdf]

Effect of the crosslinking on the rheology and cellular behavior of polymer-based 3D-multilayered scaffolds for articular cartilage restoration

Yaima Campos[a,b], *Francisco J. Sola*[a], *Amisel Almirall*[a], *Gastón Fuentes*[a,c,★], *Luis Quintanilla*[d], *Luis J. Cruz*[b], *José C. Rodríguez-Cabello*[d], *Yasuhiko Tabata*[c]

[a] Centro de Biomateriales, Universidad de La Habana, ave Universidad e/ G y Ronda, Vedado, Plaza, CP 10400, La Habana, Cuba.

[b] Department of Radiology, LUMC, The Netherlands

[c] Laboratory of Biomaterials, Department of Regeneration Science and Engineering, Institute for Frontier Life and Medical Sciences, Kyoto University, 53 Kawara-cho Shogoin, Sakyo-ku, Kyoto 606-8507, Japan

[d] Bioforge Lab, Campus Miguel Delibes, CIBER-BBN, Universidad de Valladolid, Edificio LUCIA, Paseo Belén 19, 47011, Valladolid, España

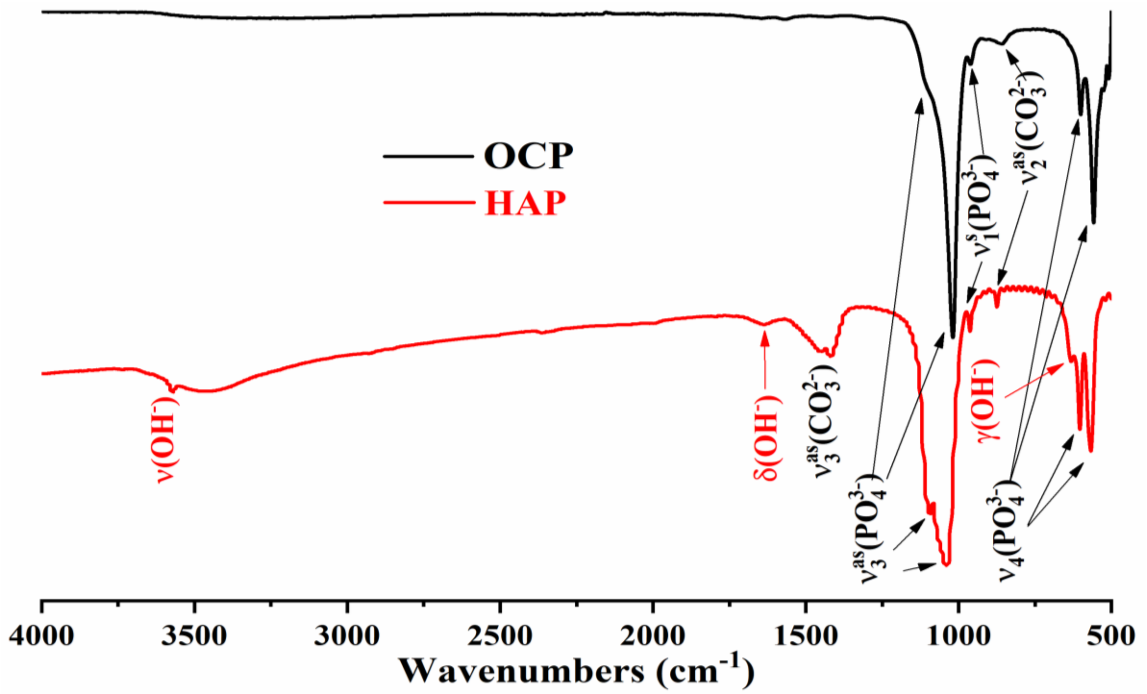

Figure S1. FTIR spectra of synthetized calcium phosphates. The main differences in the bands related with the hydroxyl groups in hydroxyapatite related to octacalcium phosphate is signalized with red color in the proper structure.

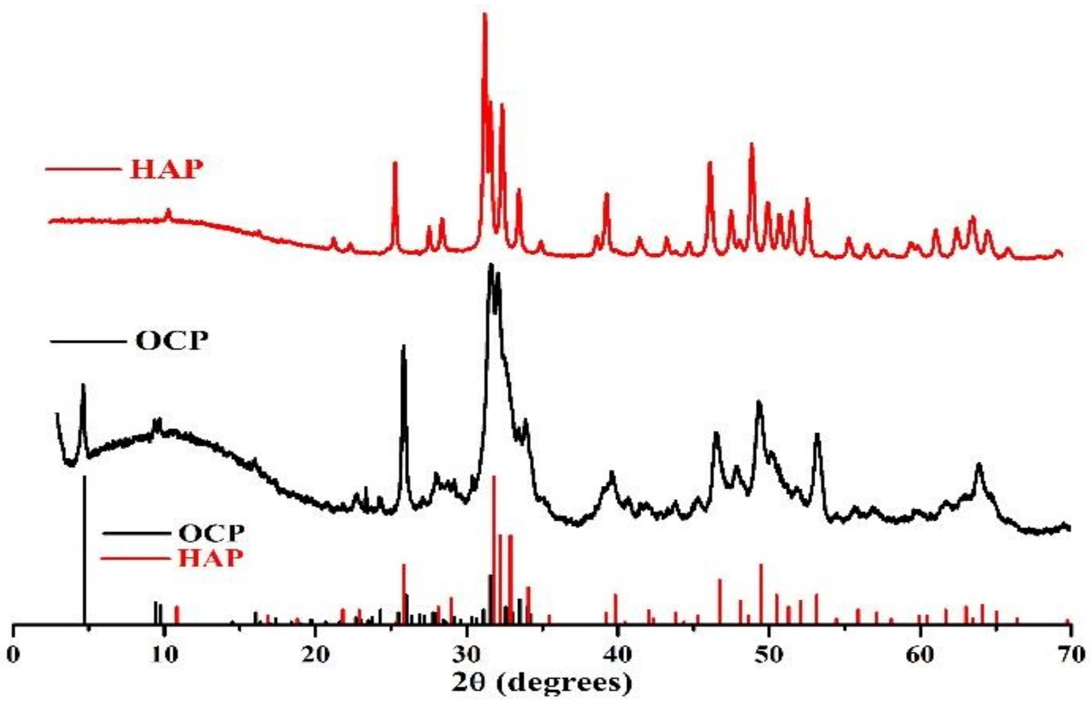

Figure S2. XRD of synthetized Ca-P materials. Bars indicated Ca-P patterns. ASTM # 26-1059 for OCP and 09-432 for HAP

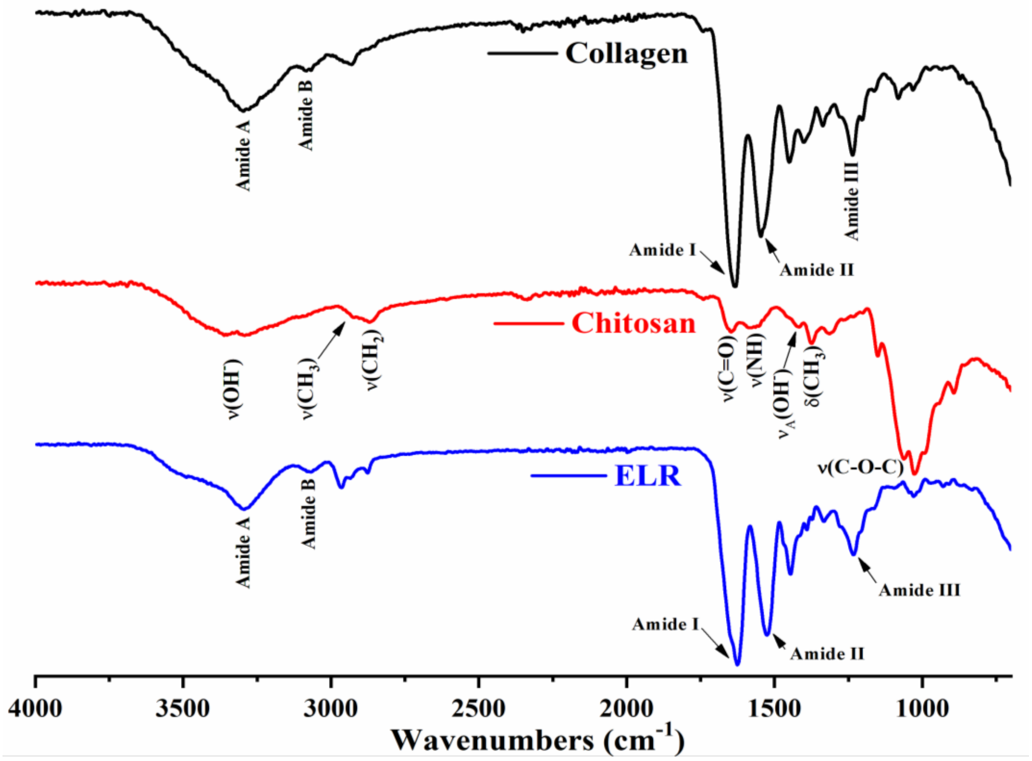

Figure S3. FTIR spectra of three polymers used in this study: COL, CHI and ELR. The very similar structure is related with the chemical structure of each one.

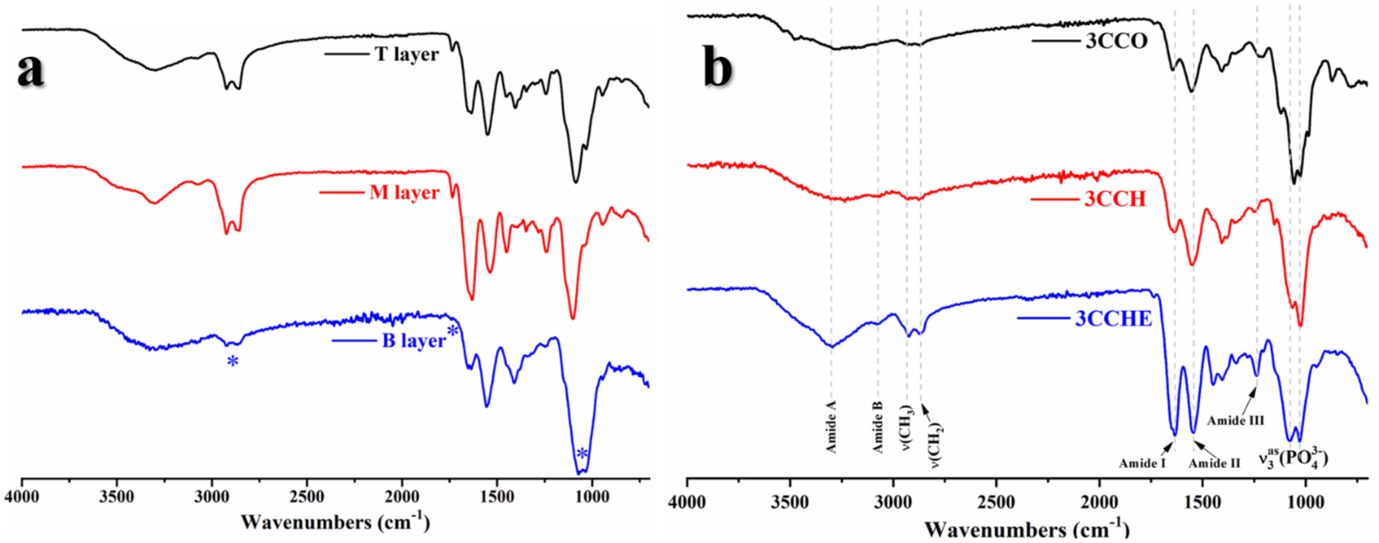

Figure S4. (a) FTIR spectra of the individual layers integrating the 3CCHE scaffold. The blue asterisk indicates areas with the main changes in the B-layer due to HAP. Each spectrum is the result of a measurement on each separate layer and (b) FTIR spectra of the scaffolds without crosslinking. To remark the increase of the bands around 1000 cm^{-1} due to addition of calcium phosphates to the scaffolds.

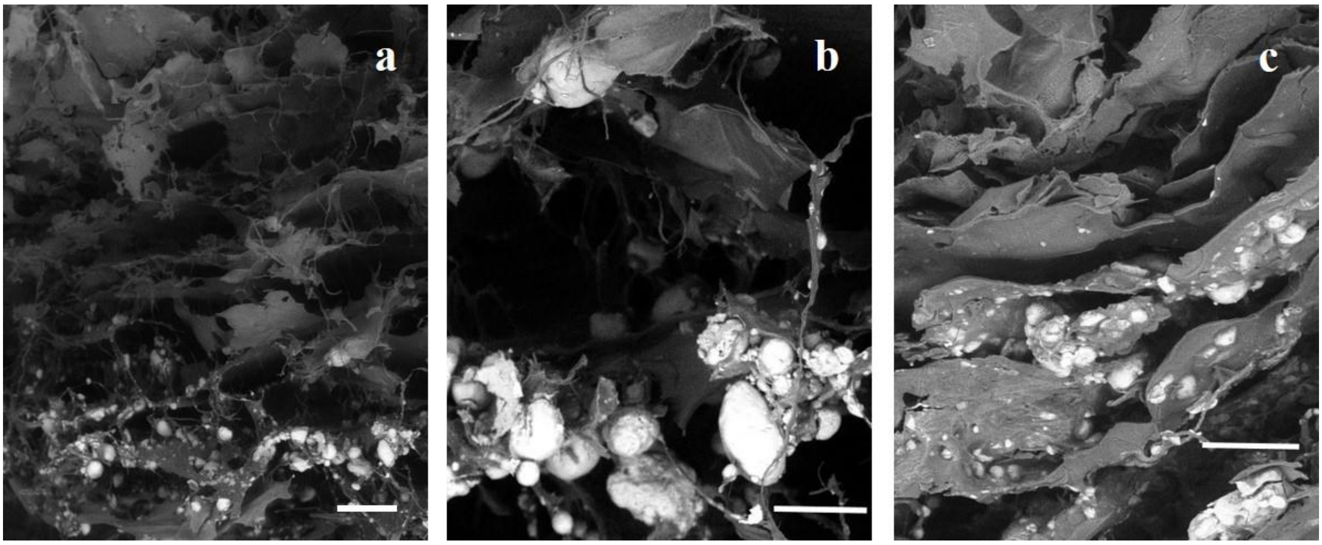

Figure S5. Micrograph of the overall structure of the 3CCHE scaffold (a, bar = 250 µm). Micrographs of the boundary between the B and M layers in the 3CCHE (b) and the 3CCHE.G (c) scaffolds. Bar = 100 µm.

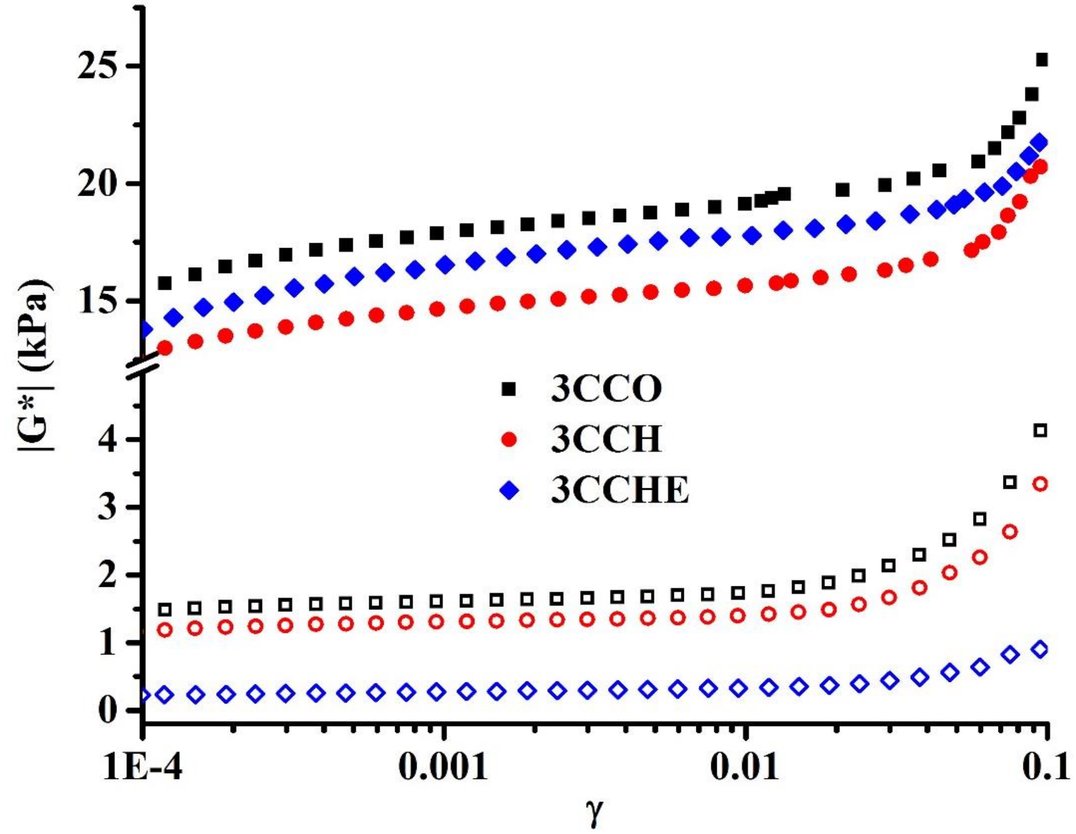

Figure S6. Evolution of complex modulus magnitude with strain amplitude for physically (open) and chemically (fill) crosslinked scaffolds

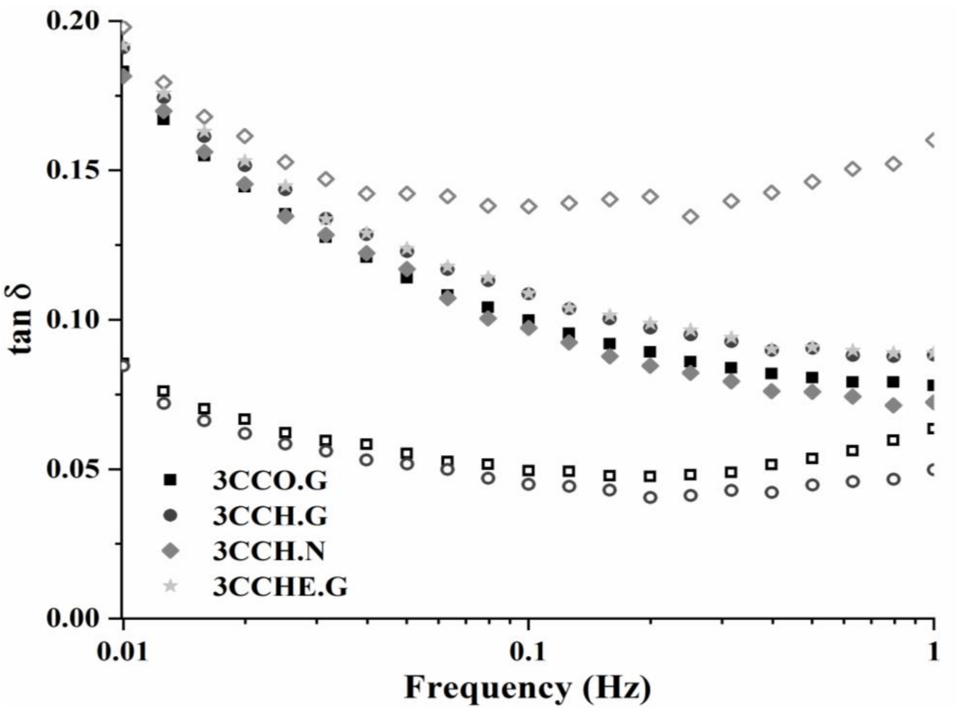

Figure S7. Loss factor for physically (open) and chemically (fill) crosslinked scaffolds. Each curve corresponds to the average of three different samples measured. Error bars have been omitted for clarity.

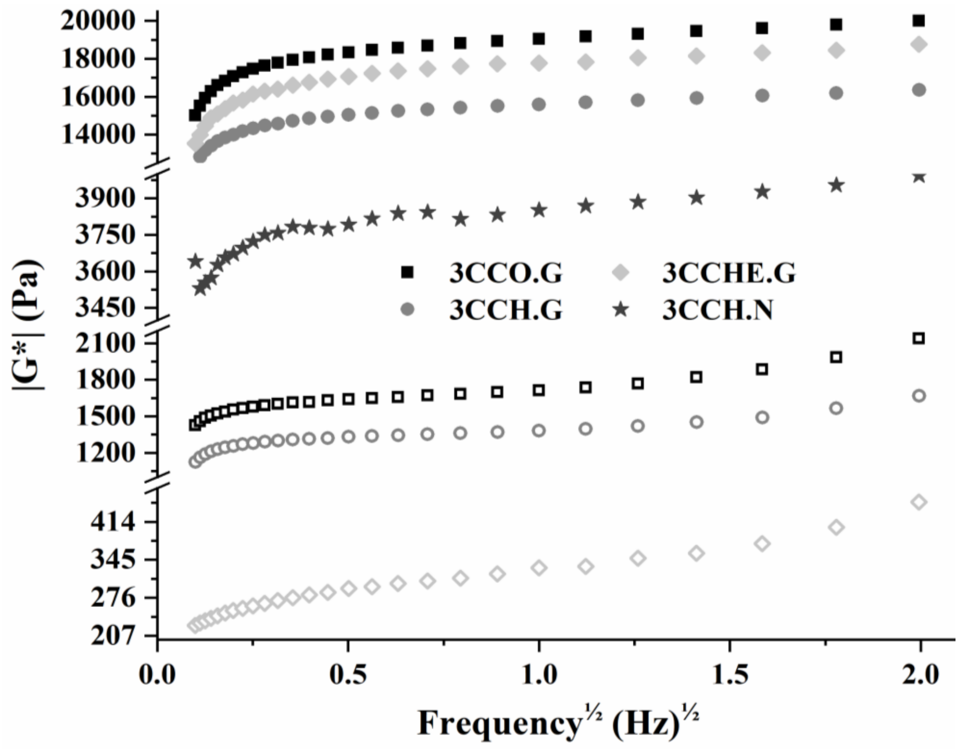

Figure S8. Complex modulus magnitude of physically (open) and chemically (fill) crosslinked scaffolds. Each curve corresponds to the average of three different samples measured. Error bars have been omitted for clarity.

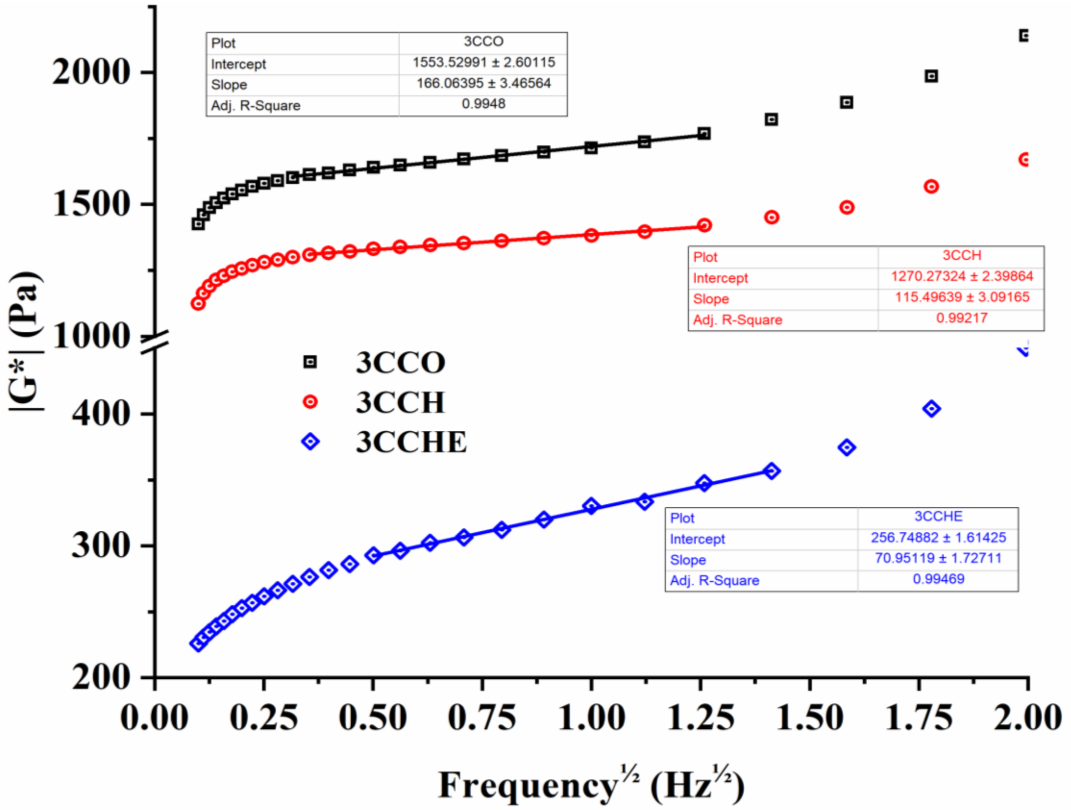

Figure S9. Evolution of complex modulus magnitude with $f^{½}$. The continuous line corresponds to the linear regression fitting in the range of 0.25-3 Hz for physically crosslinked scaffolds

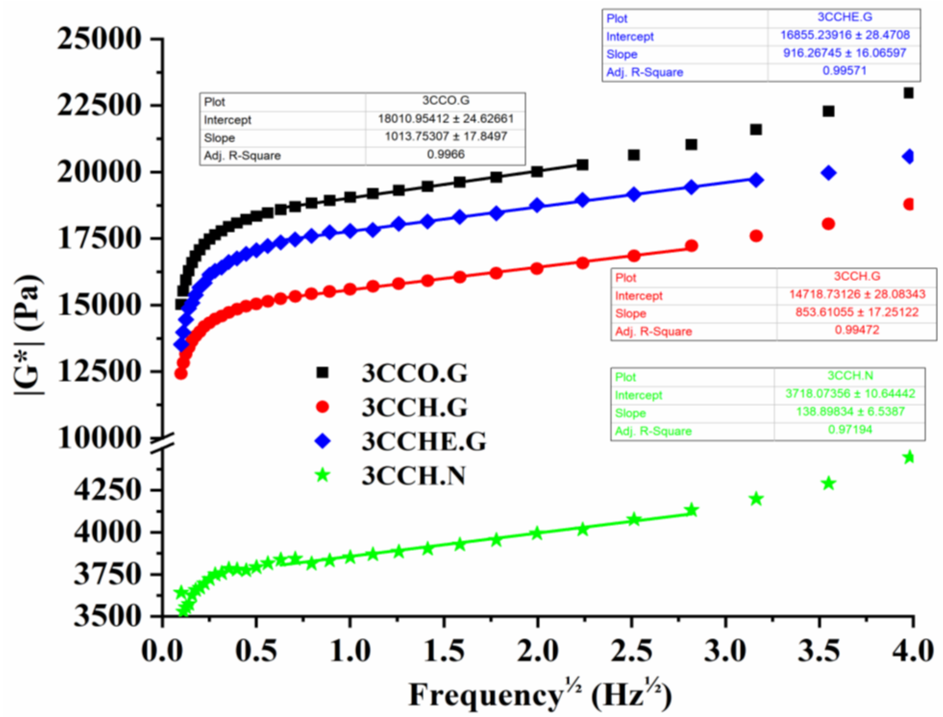

Figure S10. Evolution of complex modulus magnitude with $f^{½}$. The continuous line corresponds to the linear regression fitting in the range of 0.25-10 Hz for chemically crosslinked scaffolds

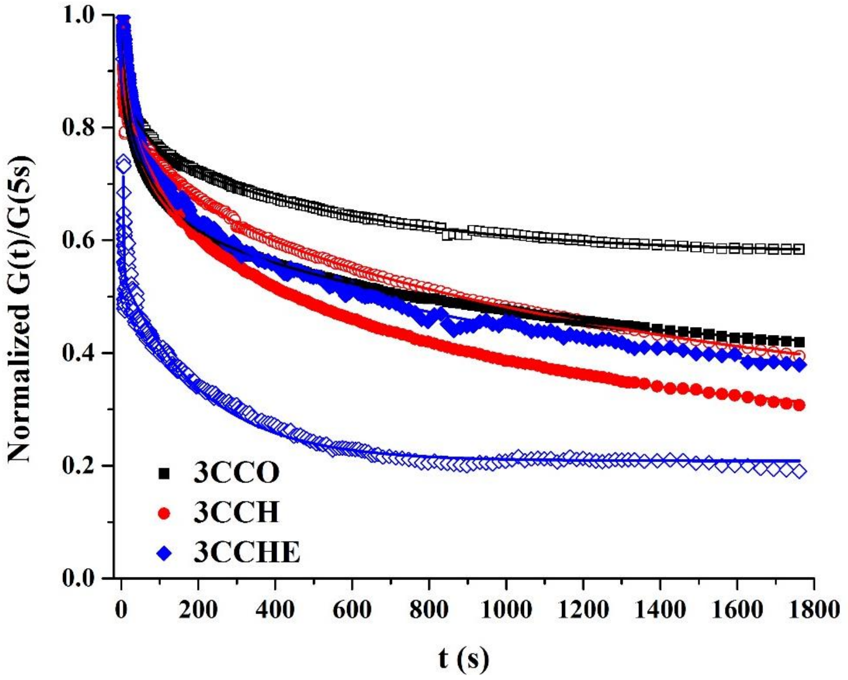

Figure S11. Normalized relaxation modulus transients. The solid line corresponds to the exponential decay fitting of third grade (according to Equation 2) for all the scaffolds (open and fill symbols correspond to physically and chemically crosslinked scaffolds respectively, with the exception of 3CCHE that was fitted to two relaxation processes
